# Anti-inflammatory and neuroprotective effects of an orally active apocynin derivative in pre-clinical models of Parkinson’s disease

**DOI:** 10.1186/1742-2094-9-241

**Published:** 2012-10-23

**Authors:** Anamitra Ghosh, Arthi Kanthasamy, Joy Joseph, Vellareddy Anantharam, Pallavi Srivastava, Brian P Dranka, Balaraman Kalyanaraman, Anumantha G Kanthasamy

**Affiliations:** 1 Department of Biomedical Sciences, Iowa Center for Advanced Neurotoxicology, Iowa State University, Ames, IA, 50011, USA; 2 Department of Biophysics, Medical College of Wisconsin, Milwaukee, WI, 53226, USA

**Keywords:** Parkinson’s disease, Oxidative stress, Neuroinflammation, Neuroprotection, Dopamine, Microglia, Diapocynin, Astrocytes

## Abstract

**Background:**

Parkinson’s disease (PD) is a devastating neurodegenerative disorder characterized by progressive motor debilitation, which affects several million people worldwide. Recent evidence suggests that glial cell activation and its inflammatory response may contribute to the progressive degeneration of dopaminergic neurons in PD. Currently, there are no neuroprotective agents available that can effectively slow the disease progression. Herein, we evaluated the anti-inflammatory and antioxidant efficacy of diapocynin, an oxidative metabolite of the naturally occurring agent apocynin, in a pre-clinical 1-methyl-4-phenyl-1,2,3,6-tetrahydropyridine (MPTP) mouse model of PD.

**Methods:**

Both pre-treatment and post-treatment of diapocynin were tested in the MPTP mouse model of PD. Diapocynin was administered via oral gavage to MPTP-treated mice. Following the treatment, behavioral, neurochemical and immunohistological studies were performed. Neuroinflammatory markers, such as ionized calcium binding adaptor molecule 1 (Iba-1), glial fibrillary acidic protein (GFAP), gp91phox and inducible nitric oxide synthase (iNOS), were measured in the nigrostriatal system. Nigral tyrosine hydroxylase (TH)-positive neurons as well as oxidative markers 3-nitrotyrosine (3-NT), 4-hydroxynonenal (4-HNE) and striatal dopamine levels were quantified for assessment of the neuroprotective efficacy of diapocynin.

**Results:**

Oral administration of diapocynin significantly attenuated MPTP-induced microglial and astroglial cell activation in the substantia nigra (SN). MPTP-induced expression of gp91phox and iNOS activation in the glial cells of SN was also completely blocked by diapocynin. Notably, diapocynin markedly inhibited MPTP-induced oxidative markers including 3-NT and 4-HNE levels in the SN. Treatment with diapocynin also significantly improved locomotor activity, restored dopamine and its metabolites, and protected dopaminergic neurons and their nerve terminals in this pre-clinical model of PD. Importantly, diapocynin administered 3 days after initiation of the disease restored the neurochemical deficits. Diapocynin also halted the disease progression in a chronic mouse model of PD.

**Conclusions:**

Collectively, these results demonstrate that diapocynin exhibits profound neuroprotective effects in a pre-clinical animal model of PD by attenuating oxidative damage and neuroinflammatory responses. These findings may have important translational implications for treating PD patients.

## Background

Parkinson’s disease (PD) is the most common neurodegenerative movement disorder, estimated to affect 1% of the population over 65 years of age. It is a chronic and progressive disease characterized predominantly by resting tremors, bradykinesia, muscular rigidity and postural instability, along with several non-motor symptoms [1]. The pathological hallmarks of PD are the depletion of striatal dopamine caused by degeneration of dopaminergic neurons in the substantia nigra (SN) region of the midbrain, appearance of cytoplasmic inclusions, known as Lewy bodies in surviving neurons of the SN, and activation of glial cells [2,3].

Although the etiologic mechanisms of PD are poorly understood, recent reports implicate brain inflammation and oxidative stress play an important role in disease pathogenesis [2,4]. Microglia and astrocytes are major mediators of neuroinflammation in PD. Several reports have demonstrated the activation of microglial cells and astroglial cells in close proximity to the damaged or dying dopaminergic neurons in SN [5]. Levels of nitrite (NO_2_^−^), a metabolite of nitric oxide (^·^NO and inducible nitric oxide synthase (iNOS) are higher in the central nervous system of human PD cases and in animal models of PD [6]. Consistent with this finding, iNOS knockout animals were resistant to 1-methyl-4-phenyl-1,2,3,6-tetrahydropyridine (MPTP)-induced dopaminergic neuronal loss in the SN [7].

One of the major sources of reactive oxygen species (ROS) in neurodegeneration is NADPH oxidase, a multimeric enzyme that generates both superoxide O_2_^−^ (O_2_ and H_2_O_2_[8]. The reaction between O_2_^−^ and ^·^NO forms peroxynitrite (ONOO^-^), another key player of dopaminergic neurodegeneration in PD. Moreover, 4-hydroxynonenal (4-HNE), an unsaturated aldehyde derived from lipid hydroperoxidase, is reported to mediate the induction of neuronal apoptosis in the presence of oxidative stress [9]. Collectively, these findings strongly suggest that mitigation of neuroinflammation and oxidative stress may be a viable neuroprotective strategy for treatment of PD.

Several inhibitors of NADPH oxidase have been tested for their anti-inflammatory and antioxidant effects in dopaminergic cells [10]. For example, apocynin (4-hydroxy-3-methoxyacetophenone), a plant-derived antioxidant, has been widely used as an NADPH oxidase inhibitor in *in vitro* and *in vivo* experimental models of PD [10-12]. However, apocynin failed to protect dopaminergic neurons against rotenone-mediated neurotoxicity in the absence of glial cells [4]. *In vivo*, apocynin has been shown to form diapocynin, a dimer of apocynin, resulting in the inhibition of NADPH oxidase [13].

Thus, in the present study, we synthesized the dimeric derivative diapocynin and tested its antioxidant and anti-inflammatory efficacies in mouse models of PD. The results presented here show that diapocynin suppresses MPTP-induced glial activation, attenuates nigral expression of proinflammatory molecules, reduces oxidative stress and protects the nigrostriatal axis after MPTP administration. Collectively, these results suggest that additional pre-clinical development of diapocynin may yield an effective neuroprotective and anti-neuroinflammatory drug capable of arresting the progression of PD.

## Methods

### Animals and treatment

Eight-week-old male C57BL/6 mice weighing 24 to 28 g were housed in standard conditions: constant temperature (22 ± 1°C), humidity (relative, 30%) and a 12 h light/dark cycle. Use of the animals and protocol procedures were approved and supervised by the Institutional Animal Care and Use Committee (IACUC) at Iowa State University (Ames, IA, USA) To assess the neuroprotective effect of diapocynin, we first used low doses of diapocynin (100 and 150 mg/kg/day) via oral gavage, but these doses showed only a moderate effect in attenuating MPTP-induced neurochemical deficits. Therefore, we used a 300 mg/kg dose of diapocynin in the subacute MPTP model of PD for detailed characterization of the neuroprotective efficacy of diapocynin. This 300 mg/kg dose was chosen based on apocynin, which has been used at a similar dose range in amyotrophic lateral sclerosis (ALS) and Alzheimer's disease mouse models [14].

In the subacute MPTP regimen, mice received 25 mg/kg/day MPTP-HCl in saline intraperitoneally for 5 consecutive days. Diapocynin was dissolved in 10% ethanol 1 day before the MPTP insult and the drug treatment continued for 11 days. Animals were subjected to measurements of inflammatory markers, neurotransmitter levels, behavioral changes and neuronal damage at various time points. Control mice received equivolumes of the vehicle solution.

In the post-treatment regimen, diapocynin was administered 3 days after the MPTP treatment. For chronic MPTP treatment, mice received 2 doses of MPTP (25 mg/kg/dose, s.c.) and 2 doses of probenecid (250 mg/kg/dose, i.p.) per week for 5 consecutive weeks. Mice received 3 doses of diapocynin (100 mg/kg/day) per week by oral gavage, and the drug treatment started 1 week before MPTP injections, continued throughout the MPTP injection period and extended for another 45 days of post-MPTP treatment. After all treatments, animals were subjected to behavioral, neurochemical and histological measurements.

### High-performance liquid chromatography (HPLC) analysis of striatal dopamine and its metabolite levels

Samples were prepared and quantified, as described previously [15,16]. On the day of analysis, neurotransmitters from striatal tissues were extracted using an antioxidant extraction solution (0.1 M perchloric acid, containing 0.05% Na_2_EDTA and 0.1% Na_2_S_2_O_5_) and isoproterenol (as internal standard). Dopamine, 3,4-dihydroxyphenyl-acetic acid (DOPAC) and homovanillic acid (HVA) were separated isocratically by a reversed-phase column with a flow rate of 0.6 ml/min. An HPLC system (ESA, Inc, Bedford, MA, USA) with an automatic sampler equipped with refrigerated temperature control (Model 542; ESA, Inc) was used for these experiments.

### HPLC/mass spectrometry (MS) analysis of diapocynin

Diapocynin from the striatum and SN was quantified using the Agilent 1100 Series LC/MS binary pump (Agilent, Santa Clara, CA, USA), PDA detector (UV diode array detector) and an autosampler. On the day of analysis, a 20 μl sample was passed through the 0.2 μm filter at the eluent flow rate of 0.25 ml/min. Negative-ion, atmospheric pressure chemical ionization was used at amplitude 1.5 volts, and manual MS/MS was done. The mobile phase used in LC/MS consisted of a gradient elution. Solvent A was 480:20:0.38 water:methanol:ammonium acetate (v/v/w) and solvent B was 20:480:0.38 water:methanol:ammonium acetate (v/v/w). The standards series were taken as 0.3 μg, 1.0 μg, 3.0 μg, 10 μg, and 30 μg. The actual molecular weight of diapocynin is 329.1 g/mol, but by breaking the molecule in MS/MS it becomes 313.9 g/mol by elimination of one methyl molecule. The retention time for the diapocynin peak was 1.9 minutes. Data were fit to a straight line by linear regression analysis using Quant analysis software (Agilent, Santa Clara, CA, USA).

### Western blotting

Mice were sacrificed 4 or 7 days after MPTP treatment, and striatum and SN tissues were dissected. Brain lysates containing equal amounts of protein were loaded in each lane and separated in a 10 to 15% SDS-polyacrylamide electrophoresis gel, as described previously [15,17]. The membranes were then incubated with primary antibodies (anti-TH (Chemicon, Temecula, CA, USA), anti-Iba-1 (Abcam, Cambridge, UK), anti-GFAP (Chemicon), anti-iNOS (Santa Cruz Biotechnology, Santa Cruz, CA, USA), anti-gp91phox (Abcam), anti-3NT (Chemicon) and anti-4-HNE (R&D Systems, Minneapolis, MN, USA)). Next, membranes were incubated with Alexa Fluor 680 goat anti-mouse or Alexa Fluor 680 donkey anti-goat (Invitrogen, Carlsbad, CA, USA) or IRDye 800 donkey anti-rabbit (Rockland, Gilbertsville, PA, USA) secondary antibodies. To confirm equal protein loading, blots were reprobed with a β-actin antibody (Sigma-Aldrich, St Louis, MO, USA) at 1:10000 dilution. Western blot images were captured with a LI-COR Odyssey machine (LI-COR, Lincoln, NE, USA). The Western blot bands were quantified using ImageJ software (National Institutes of Health (NIH), Bethesda, MD, USA).

### Immunohistochemistry

One day after the last MPTP treatment, mice were perfused with 4% paraformaldehyde (PFA) and post-fixed with PFA and 30% sucrose, respectively. Next, fixed brains were cut into 30 μm sections and were incubated with primary antibodies (anti-Iba-1 (Abcam), anti-GFAP (Chemicon), anti-iNOS (Santa Cruz Biotechnology), anti-3NT (Chemicon), anti-gp91phox (Abcam), anti-TH (Chemicon) and anti-4HNE (R&D)) overnight at 4°C. Appropriate secondary antibodies (Alexa Fluor 488 or 594 or 555; Invitrogen) were used, followed by incubation with 10 μg/ml Hoechst 33342 (Invitrogen) for 5 minutes at room temperature to stain the nucleus. Sections were viewed under a Nikon inverted fluorescence microscope (Model TE-2000U; Nikon, Tokyo, Japan); images were captured with a SPOT digital camera (Diagnostic Instruments, Inc, Sterling Heights, MI, USA).

### 3,3'-Diaminobenzidine (DAB) immunostaining and stereological counting

DAB immunostaining was performed in striatum and SN sections, as described previously [3,18]. Briefly, 30 μm sections were incubated with either anti-TH antibody (Calbiochem, Billerica, MA, USA; rabbit anti-mouse, 1:1800 dilution) or anti-Iba-1 (Abcam; goat anti-mouse, 1:1000 dilution) or anti-GFAP (Chemicon; mouse anti-mouse, 1:1000 dilution) antibody, followed by incubation with biotinylated anti-rabbit or goat or mouse secondary antibody. Total numbers of tyrosine hydroxylase (TH)-positive neurons in SN were counted stereologically with Stereo Investigator software (MBF Bioscience, Williston, VT, USA), using an optical fractionator [16,19].

### Fluoro-Jade B (FJB) and TH double labeling

On the day of staining, sections were incubated with anti-TH antibody (Chemicon), followed by Alexa Fluor 568 donkey anti-mouse (Invitrogen) secondary antibody. Then FJB staining was done on the same sections by the modified FJB stain protocol, including incubation in 0.06% potassium permanganate for 2 minutes and 0.0002% FJB stain for 5 minutes. Sections were viewed under a Nikon inverted fluorescence microscope (Model TE-2000U; Nikon) and images were captured with a SPOT digital camera (Diagnostic Instruments, Inc).

### Behavioral measurements

An automated device (Model RXYZCM-16; Accuscan, Columbus, OH, USA) was used to measure the spontaneous activity of mice. The activity chamber was 40 × 40 × 30.5 cm, made of clear Plexiglas and covered with a Plexiglas lid with holes for ventilation. Data were collected and analyzed by a VersaMax Analyzer (Model CDA-8; AccuScan). Before any treatment, mice were placed inside the infrared monitor for 10 minutes daily for 3 consecutive days to train them. Five days after the last MPTP injection, open field and rotarod experiments were conducted. Locomotor activities were recorded for 10 minute test sessions. A speed of 20 rpm was used in the rotarod experiment. Mice were given a 7 to 10 minute rest interval to prevent stress and fatigue.

### Data analysis

Data analysis was performed using Prism 4.0 software (GraphPad Software, Inc, San Diego, CA, USA). Raw data were first analyzed using one-way analysis of variance and then Tukey’s post-test was performed to compare all treatment groups. Differences with *P <*0.05 were considered significant.

## Results

### Diapocynin enters brain and attenuates MPTP-induced striatal neurotransmitter depletion

**Figure 1:**
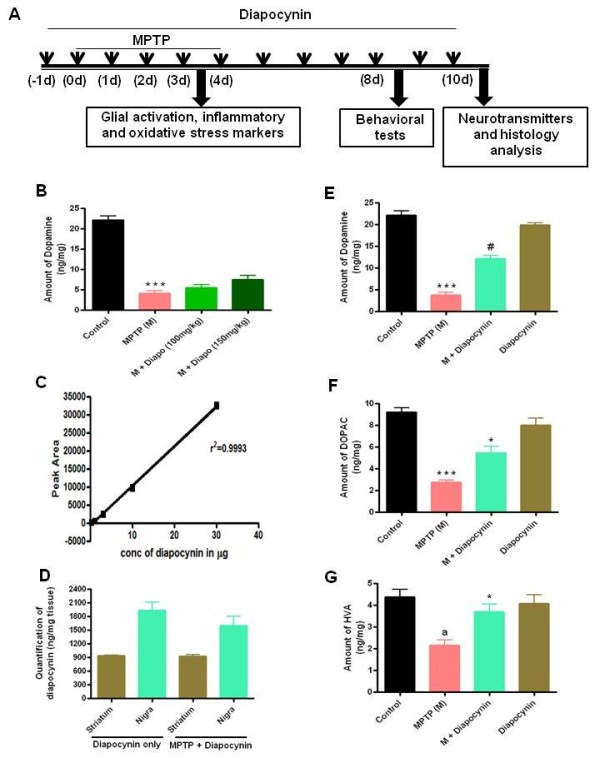
**Diapocynin enters brain and attenuates MPTP-induced depletion of neurotransmitters.** (**A**) Treatment schedule of MPTP-injected mice with diapocynin. (**B**) Mice were treated with two doses of diapocynin (100 and 150 mg/kg/day) and seven days after the last dose of MPTP treatment, striatal dopamine level was measured using high-performance liquid chromatography (HPLC). Quantification of diapocynin in the substantia nigra (SN) and striatum of mice. Mice were administered diapocynin (300 mg/kg/day) by oral gavage 24 h before MPTP treatment, and co-treatment with MPTP (25 mg/kg/day) was continued for 5 days, and post-treatment with MPTP (25 mg/kg/day) lasted 6 days. Seven days after the last injection of MPTP, SN and striatum were dissected out and quantified for diapocynin. (**C**) Standard curve of diapocynin standards ranging from 0.3 μg to 30 μg. (**D**) Quantification of diapocynin in SN and striatum. Seven days after the last MPTP treatment, striatal (**E**) dopamine, (**F**) 3,4-dihydroxyphenyl-acetic acid (DOPAC) and (**G**) homovanillic acid (HVA) were measured by HPLC. Data are means ± SEM of eight to ten mice per group. ^***^P <0.001 versus the control group; ^*^P <0.05 vs the MPTP group; ^#^*P* <0.01 vs the MPTP group; ^a^*P* <0.01 vs the control group.

Male C57BL/6 mice were treated with diapocynin by oral gavage daily and then inflammatory, neurochemical, behavioral and histological studies were performed at various time intervals, as depicted in Figure 1A. To assess the neuroprotective effect of diapocynin, we first used two lower doses of diapocynin (100 and 150 mg/kg/day) and measured the dopamine level from striatum (Figure 1B). Both 100 and 150 mg/kg/day doses of diapocynin afforded some protection against MPTP-induced striatal dopamine loss, but it was not significant (Figure 1B). Therefore, we increased the dosage of diapocynin to 300 mg/kg/day and then evaluated the neuroprotective effects. Before determining the neuroprotective properties of diapocynin, the ability of diapocynin to cross the blood-brain barrier was examined. The LC/MS results showed a significant accumulation of diapocynin in the SN and striatum of the brain following the oral gavage treatment of diapocynin (300 mg/kg/day) (Figure 1C,D).

Following 300 mg/kg/day diapocynin treatment, mice were sacrificed and brains were processed for HPLC neurochemical analysis (Figure 1E,G). We quantified levels of dopamine and its metabolites, DOPAC and HVA, in striatum following diapocynin treatment. As shown in Figure 1E, MPTP injection led to a >75% reduction in striatal dopamine levels compared with the striata of control mice. Remarkably, diapocynin treatment significantly protected against MPTP-induced striatal dopamine depletion (Figure 1E). Diapocynin treatment also significantly restored DOPAC and HVA levels in MPTP-treated mice (Figure 1F,G).

In order to determine whether diapocynin interferes with the toxic metabolic conversion of MPTP to MPP^+^ by MAO-B, we measured the level of MPP^+^ in striatum 3 h after the final MPTP injection. We found that diapocynin had no effect on striatal levels of MPP^+^ (MPTP mice, 1860 ± 569 ng/g; MPTP plus diapocynin mice, 1775 ± 586 ng/g). Next, we also tested whether diapocynin treatment alone alters neurochemical levels in the striatum. As shown in Figure 1E,G, oral administration of diapocynin (300 mg/kg/day) alone for 12 days did not alter striatal dopamine levels. Also, diapocynin treatment did not produce behavioral abnormalities (data not shown).

### Diapocynin inhibits MPTP-induced glial activation in the SN

**Figure 2:**
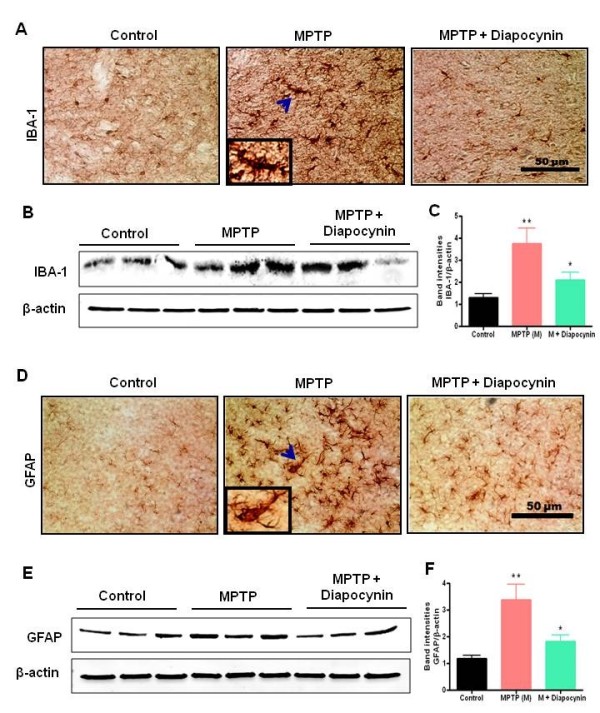
**Diapocynin inhibits activation of microglia and astrocytes in the substantia nigra (SN) of MPTP-treated mice.** (**A**) 3,3'-Diaminobenzidine (DAB) immunostaining of Iba-1 in SN. (**B**) Representative Western blots illustrating the expression of Iba-1 in SN. (**C**) Bar graph showing quantitative densitometric analysis of Iba-1/β-actin ratio ± SEM in SN of 6 mice per group. (**D**) DAB immunostaining of glial fibrillary acidic protein (GFAP) in SN region of ventral midbrain. (**E**) Representative Western blots illustrating the expression of GFAP in SN. (**F**) Bar graph showing mean Western blot GFAP/β-actin ratios ± SEM in SN of 6 mice per group. Images were captured at 30× magnification. ^**^P <0.01 vs the control group; ^*^P <0.05 vs the MPTP group.

Next, we determined if diapocynin protected against MPTP-induced microglial activation and astrogliosis. As shown in Figure 2, a significant increase in the expression of ionized calcium binding adaptor molecule 1 (Iba-1) and glial fibrillary acidic protein (GFAP) in SN of the MPTP-treated group was observed. DAB immunostaining of Iba-1 in MPTP nigral brain tissue showed an increased number of amoeboid-shaped microglial cells with thick processes, indicative of active microgliosis (Figure 2A). DAB immunostaining of GFAP in MPTP-treated mice showed excessive astrogliosis, as measured by increased size and processes thickness in GFAP-positive cells (Figure 2D).

Importantly, diapocynin strongly inhibited MPTP-induced microgliosis, as demonstrated by the very few Iba-1-positive microglial cells in the drug-treated group (Figure 2A). Also, diapocynin significantly decreased MPTP-induced increases in GFAP-positive astroglial cells in the mouse SN (Figure 2D). Western blot analysis for Iba-1 and GFAP also revealed that diapocynin suppresses nigral Iba-1 (Figure 2B and C) and GFAP (Figure 2E,F) protein expression in MPTP-treated mice. Together, these data suggest that diapocynin significantly attenuates glial cell activation in the nigral regions of the MPTP mouse model of PD.

### Diapocynin inhibits MPTP-induced iNOS expression in mouse SN

**Figure 3:**
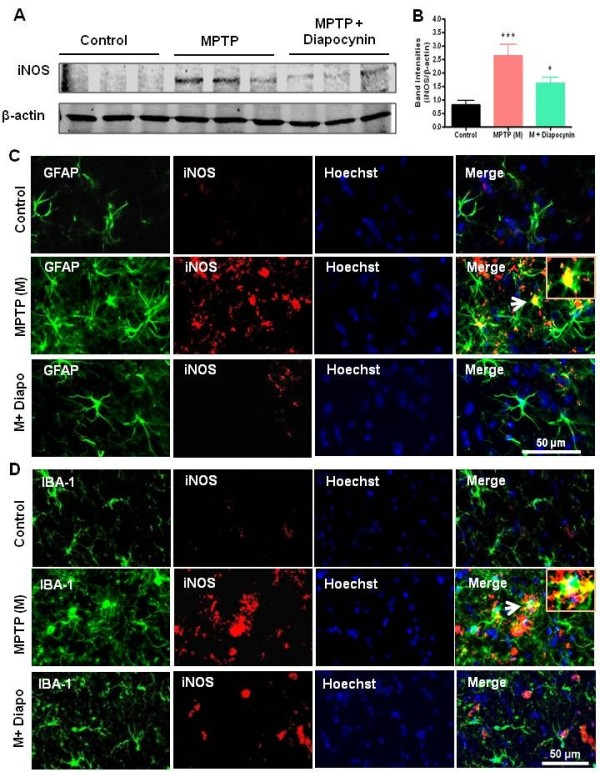
**Diapocynin attenuates inducible nitric oxide synthase (iNOS) expression in the substantia nigra (SN) of MPTP-treated mice.** (**A**) Representative Western blots illustrating the expression of iNOS in SN. (**B**) Bar graph showing means of Western blot iNOS/β-actin ratios ± SEM in SN of 6 mice per group. (**C**) Double labeling of glial fibrillary acidic protein (GFAP) and iNOS, and (**D**) Iba-1 and iNOS in the SN region of ventral midbrain. Images were captured at 30× and 60× magnification. ^***^P <0.001 vs the control group; ^*^P <0.05 vs the MPTP group.

iNOS, a key proinflammatory enzyme, is typically elevated in disease conditions [3]. We observed a marked increase in the expression of iNOS in the SN of MPTP-treated mice as compared to control mice (Figure 3A,B). However, diapocynin attenuated MPTP-induced expression of iNOS protein (Figure 3A,B). Additionally, immunofluorescence analysis for iNOS in the SN sections shows that MPTP treatment led to a marked increase in nigral iNOS protein expression, and that iNOS co-localized with GFAP-positive astrocytes and Iba-1-positive microglial cells (Figure 3C,D). Consistent with its inhibitory effect on glial cell activation, diapocynin suppressed MPTP-induced expression of iNOS (Figure 3C,D). These results demonstrate that diapocynin effectively suppresses the expression of the proinflammatory molecule iNOS *in vivo* in the SN of MPTP-treated mice.

### Diapocynin attenuates MPTP-induced activation of microglial NADPH oxidase

**Figure 4:**
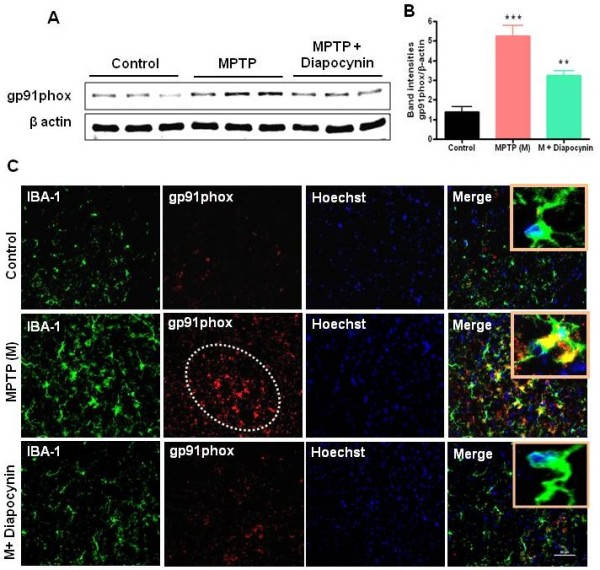
**Diapocynin attenuates NADPH oxidase mediated inflammatory responses in the substantia nigra (SN) of MPTP-treated mice.** (**A**) Representative Western blots illustrating the expression of gp91phox (membrane-bound subunit of NADPH oxidase) in SN. (**B**) Bar graph showing mean Western blot gp91phox/β-actin ratios ± SEM in SN of 6 mice per group. (**C**) SN tissue sections were double labeled for gp91phox and Iba-1. Images were captured at 20× and 60× (insets) magnification. The SN zone is outlined in white dots. Inset pictures demonstrated colocalization of Iba-1 and gp91phox. ^***^P <0.001 vs the control group; ^*^P <0.05 vs the MPTP group.

NADPH oxidase is a major source of ROS in the brain. Recent findings suggest that NADPH oxidase-induced oxidative stress plays a central role in nigral dopaminergic neurodegeneration in PD patients and animal models [20]. Western blot analysis showed an increased expression of gp91phox, a key subunit of NADPH oxidase, after MPTP injection compared to weak expression in saline-treated mice (Figure 4A,B). Robust gp91phox immunoreactivity was seen specifically in larger cells with thick, shorter ramifications in the SN of MPTP-treated mice (Figure 4C). Double immunolabeling studies confirmed that gp91phox immunoreactivity appeared to co-localize with Iba-1-positive microglia (Figure 4C, middle panel). Diapocynin treatment attenuated MPTP-induced gp91phox protein expression in the Iba-1-positive microglial cells in the SN (Figure 4C). In the MPTP model of PD, gp91phox does not co-localize with either astrocytes or dopaminergic neurons [20]. These results suggest that diapocynin effectively blocks NADPH oxidase expression in response to MPTP.

### Diapocynin inhibits formation of nitrotyrosine and hydroxynonenal in the nigral dopaminergic neurons of MPTP-treated mice

**Figure 5:**
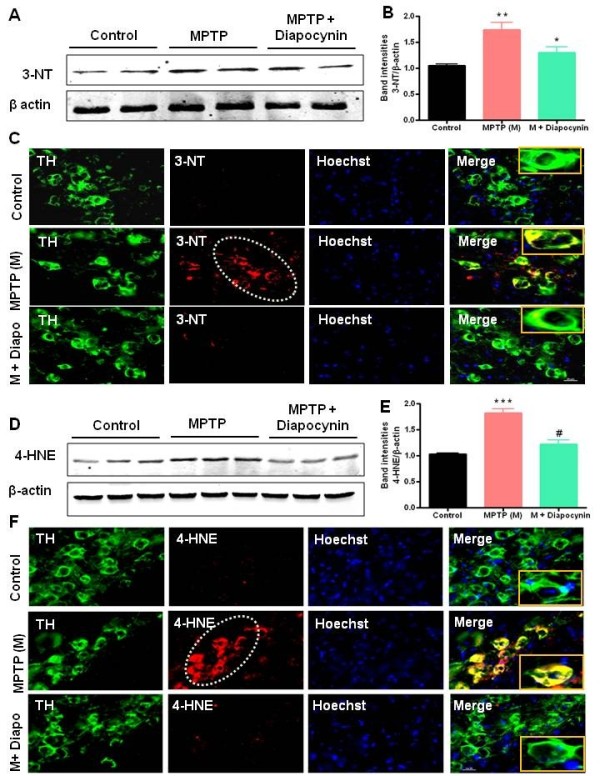
**Diapocynin inhibits the formation of 3-nitrotyrosine (3-NT) and 4-hydroxynonenal (4-HNE) in the substantia nigra (SN) of MPTP-treated mice.** (**A**) Representative Western blots illustrating the expression of 3-NT in SN. (**B**) Bar graph showing mean Western blot 3-NT/β-actin ratios ± SEM in SN of 6 mice per group. (**C**) Double labeling of tyrosine hydroxylase (TH) and 3-NT in SN region of ventral midbrain. (**D**) Representative Western blots illustrating the expression of 4-HNE in SN. (**E**) Bar graph showing mean Western blot 4-HNE/β-actin ratios ± SEM in SN of 6 mice per group. (**F**) Double labeling of TH and 4-HNE in SN region of ventral midbrain. Images were captured at 60× magnification. The SN zone is outlined in white dots. Inset pictures demonstrated colocalization of TH and 3-NT/4-HNE. ^***^P <0.001 vs the control group; ^**^P <0.01 vs the control group; ^*^P <0.05 vs the MPTP group; ^#^*P* <0.001 vs the MPTP group.

3-Nitrotyrosine (3-NT) has been widely used as a marker of nitric oxide-dependent oxidative stress [21]. Western blot analysis demonstrated increased expression of 3-NT modified proteins in the SN of MPTP-treated mice, while diapocynin treatment significantly suppressed MPTP-induced 3-NT levels (Figure 5A,B). Further confirmation came from immunolabeling of 3-NT in the SN sections. In sections from MPTP-treated mice, a dramatic increase in the expression of 3-NT, specifically in the SN region of the ventral midbrain, was observed. Notably, 3-NT co-localized (yellow color) with TH-positive dopaminergic neurons (Figure 5C). However, 3-NT expression was observed in very few TH-positive dopaminergic neurons in the MPTP and diapocynin co-treated animals (Figure 5C). These results strongly suggest that diapocynin inhibits nitration of TH-positive dopaminergic neurons in the nigra during neurotoxic insult.

In addition to protein nitration, MPTP treatment significantly increased oxidative damage in the nigral regions, as measured by 4-HNE Western blot analysis (molecular weight of 68kDa) (Figure 5D,E). However, diapocynin strongly inhibited MPTP-induced expression of this unsaturated aldehyde in the SN (Figure 5D,E). Consistent with these findings, immunofluorescence analysis of the MPTP-treated SN sections showed a dramatic increase in 4-HNE formation in the dopaminergic neurons, as evidenced by TH and 4-HNE double immunolabeling (Figure 5F). Interestingly, diapocynin treatment abolished MPTP-induced 4-HNE generation in dopaminergic neurons (Figure 5F). These results suggest that diapocynin mitigates oxidative damage in nigral dopaminergic neurons following MPTP neurotoxicity.

### Diapocynin protects against MPTP-induced neurodegeneration

**Figure 6:**
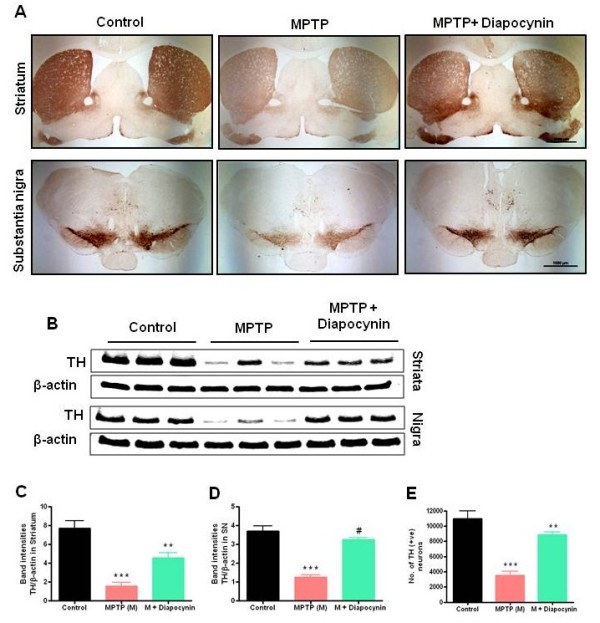
**Diapocynin protects nigrostriatum in MPTP-treated mice.** (**A**) Seven days after MPTP treatment mice were sacrificed, tyrosine hydroxylase (TH)-3,3'-diaminobenzidine (DAB) immunostaining in the striatum and substantia nigra (SN) regions was performed (2× magnifications). (**B**) Representative Western blots illustrating the expression of TH in SN and striatum. Bar graphs showing mean Western blot TH/β-actin ratios ± SEM in (**C**) striatum and (**D**) SN of 6 mice per group. (**E**) Stereological counts of TH-positive dopaminergic neurons in the SN of ventral midbrain. Data are means ± SEM of 8 to 10 mice per group. ^***^P <0.001 vs the control group; ^**^P <0.01 vs the MPTP group; ^#^*P* <0.001 vs the MPTP group.

Next, we determined whether diapocynin protects the dopaminergic neurons against MPTP toxicity. MPTP treatment led to degeneration of TH-positive dopaminergic neurons and terminals in the SN and striatum (Figure 6A, 2× magnification). Higher magnified 10× pictures (Additional file 1: Figure S1A) clearly demonstrated loss of neurons in substantia nigra pars compacta (SNpc), substantia nigra lateralis (SNl) and substantia nigra reticularis (SNr) regions of the nigral tract. Additionally, stereological counting of TH-positive neurons in SN of MPTP-treated mice also showed > 60% reduction (Figure 6E).

Interestingly, diapocynin treatment improved MPTP-induced damage of nigral TH-positive neurons and striatal TH terminals (Figure 6A,E and Additional file 1: Figure S1A). Consistent with these findings, Western blot of TH in nigra and striatum also showed significantly decreased TH protein levels in MPTP-treated mice (Figure 6B,C,D). However, orally administered diapocynin significantly prevented loss of nigral and striatal TH in MPTP-treated mice (Figure 6B,C,D). Further assessment of degenerating neurons in the nigral regions by FJB staining revealed that diapocynin-treated MPTP mice had fewer FJB-positive cells than untreated MPTP mice (Additional file 1: Figure S1B). Collectively, these results suggest that diapocynin is neuroprotective in the MPTP mouse model of PD.

### Diapocynin improves locomotor activities in MPTP-injected mice

**Figure 7:**
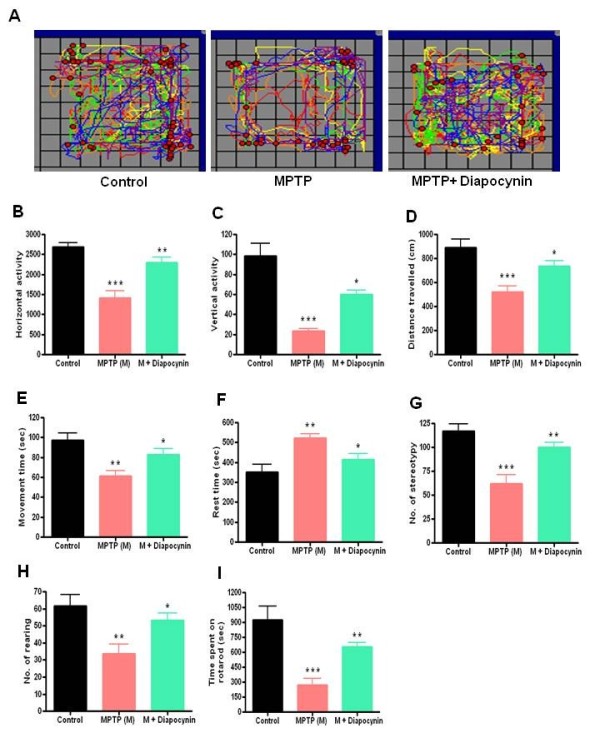
**Diapocynin improves motor function in the MPTP-injected mice.** (**A**) VersaPlot map showing moving track of mice. VersaMax data showing (**B**) horizontal activity, (**C**) vertical activity, (**D**) distance travelled (cm); (**E**) movement time (sec); (**F**) rest time (sec), (**G**) number of stereotypies, (**H**) number of rearings and (**I**) time spent on rotarod (sec) at rod speed 20 rpm. Data are means ± SEM of 8 to 10 mice per group. ^***^P <0.001 vs the control group; ^**^P <0.01 vs the MPTP group; ^*^P <0.05 vs the MPTP group; ^#^*P* <0.01 vs the control group.

To assess the effectiveness of diapocynin against motor deficits induced by MPTP, we measured various motor performance parameters using VersaMax infrared computerized activity monitoring system and a rotarod instrument (Accuscan). Behavioral function was assessed 4 days after the last dose of MPTP treatment. Representative motor activity maps of movement of saline-treated control, MPTP and MPTP plus diapocynin-treated mice are shown in Figure 7A. As expected, MPTP drastically decreased movement in all directions. Diapocynin treatment dramatically improved locomotion in the MPTP plus diapocynin-treated group. Further analysis of locomotor activity data revealed that subacute MPTP treatment markedly decreased horizontal activity (Figure 7B), vertical activity (Figure 7C), total distance travelled (Figure 7D), movement time (Figure 7E), observed stereotypies (Figure 7G), observed rearings (Figure 7H) and rotarod performances at 20 rpm speed (Figure 7I), consistent with our previous observations [16]. Additionally, rest time was increased in the MPTP-treated mice (Figure 7F). Notably, diapocynin treatment significantly restored MPTP-induced locomotor and motor co-ordination impairments for every endpoint measured (Figure 7B,C,D,E,F,G,H,I).

### Post-treatment with diapocynin rescues striatal neurotransmitter depletion in MPTP-treated mice

**Figure 8:**
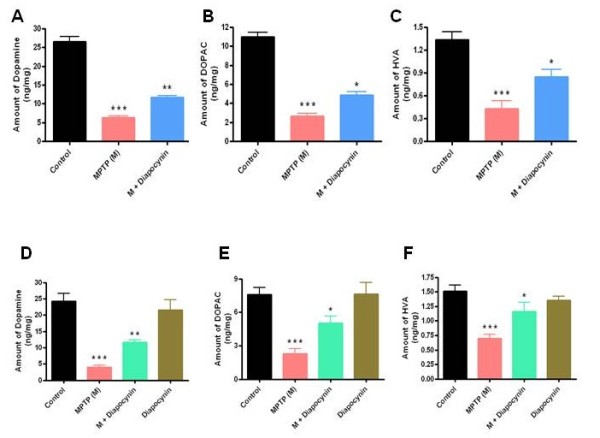
**Diapocynin suppresses disease progression in the subacute MPTP mouse model of Parkinson’s disease (PD) and protects striatal neurotransmitter depletion in the chronic MPTP mouse model of PD.** For disease progression study, mice were treated with MPTP (25 mg/kg/day) for 5 days. Diapocynin (300 mg/kg/day) treatment started on the 4th day of MPTP injection and continued for another 8 days. Mice were sacrificed 1 day after the last dose of diapocynin and striatal (**A**) dopamine, (**B**) 3,4-dihydroxyphenyl-acetic acid (DOPAC) and (**C**) homovanillic acid (HVA) were measured by high-performance liquid chromatography (HPLC). For chronic treatment, mice were treated with 2 doses of MPTP (25 mg/kg/day, s.c.) and 2 doses of probenecid (250 mg/kg/day, i.p.) per week for 5 weeks. Control mice received only saline. One week prior to MPTP/probenecid treatment, one group of mice received 3 doses of diapocynin (100 mg/kg/day, gavage) and this treatment continued for 12 consecutive weeks. Another batch of mice received 3 doses of diapocynin (100 mg/kg/day, gavage) in a week for consecutive 12 weeks. Immediately after treatment, mice were sacrificed and striatal (**D**) dopamine, (**E**) DOPAC and (**F**) HVA were measured by HPLC. Data are means ± SEM of 8 to 10 mice per group. ^***^P <0.001 vs the control group; ^**^P <0.01 vs the MPTP group; ^*^P <0.05 vs the MPTP group.

Following characterization of the neuroprotective effect of diapocynin in the typical subacute MPTP model, we further examined whether diapocynin can intervene the on-going degenerative processes in a post-treatment paradigm. In order to test the efficacy of diapocynin post-treatment, mice were treated with MPTP at a dose of 25 mg/kg/day for 3 days followed by co-treatment with MPTP and diapocynin (300 mg/kg/day) for 2 days. Mice also received another 6 doses of diapocynin (300 mg/kg/day) and were sacrificed for neurotransmitter analysis. As evident from Figure 8A,B,C, we observed significantly reduced striatal dopamine (75%), DOPAC (73%) and HVA (70%) in MPTP-treated mice. Importantly, diapocynin post-treatment showed a reduction of 52% of dopamine (Figure 8A), 50% of DOPAC (Figure 8B) and 40% of HVA (Figure 8C). Thus, these results suggest that diapocynin slows the progression of dopaminergic neurodegeneration in a post-treatment MPTP mouse model of PD.

### Diapocynin halts the disease progression in a chronic MPTP mouse model

Although the subacute MPTP model is very efficient for drug screening and elucidating molecular mechanisms, it does not recapitulate the chronic progression of degenerative processes associated with PD. In our experiment, we measured whether diapocynin efficiently protected nigrostriatum in a chronic MPTP model (Figure 8D,E,F). As expected, chronic MPTP administration led to approximately 80% loss of dopamine (Figure 8D), 60% loss of DOPAC (Figure 8E) and 70% loss of HVA (Figure 8F). Consistent with observations in the subacute MPTP model, diapocynin significantly restored dopamine (*P* <0.01; Figure 8D), DOPAC (*P* <0.05; Figure 8E) and HVA (*P* <0.05; Figure 8F) in striatum, demonstrating that oral diapocynin treatment can slow the progressive neurodegenerative process in the nigrostriatal dopaminergic system.

## Discussion

Neuroinflammation and oxidative stress are now well recognized as key pathophysiological events contributing to the progressive loss of nigral dopaminergic neurons in PD [2-4]. However, an effective neuroprotective therapy to halt the progression of the disease is not available. Here, we report the anti-inflammatory and antioxidative properties of a synthetic analog of apocynin in the MPTP mouse model of PD. Recent studies have shown conversion of apocynin to diapocynin (apocynin dimer) *in vivo,* which prevents the assembly and activation of the NADPH oxidase complex [13]. Additionally, diapocynin is 13-fold more lipophilic than apocynin [22]. Here, we show that diapocynin inhibits MPTP-induced activation and expression of both iNOS and gp91phox in activated glial cells, suggesting that diapocynin has anti-inflammatory properties against neurotoxic stress. Diapocynin also attenuates the formation of ONOO^-^ and 4-HNE in dopaminergic neurons in response to various stimuli, further confirming the antioxidant properties of this compound.

Importantly, diapocynin also protects against MPTP-induced motor deficits, striatal neurotransmitter depletion and nigrostriatal degeneration. Furthermore, diapocynin is effective in post-treatment paradigms as well as in chronic neurodegenerative models of PD. Derivatives of natural compounds, such as diapocynin, are a key translational approach for the development of therapies for PD. To our knowledge, this is the first report showing anti-inflammatory, antioxidative and neuroprotective properties of a novel apocynin derivative in animal models of PD.

NADPH oxidase has emerged as a major source of oxidative stress in the brain, particularly in neurodegenerative disorders, such as PD, Alzheimer's disease, ALS and multiple sclerosis [23]. Apocynin has been shown to inhibit NADPH oxidase, which generates ROS during inflammatory processes [10]. Although the mechanism of inhibition of apocynin is not clear, it is thought to prevent the recruitment of cytosolic NADPH oxidase subunit p47phox to the membrane, thereby inhibiting NADPH oxidase activity. Apocynin has been shown to attenuate superoxide formation and oxidative stress *in vivo* as well as reduce acute inflammation in lung and spinal cord [24-26]. Furthermore, apocynin administered at a dose of 300 mg/kg/day protects against oxidative damage induced by cerebral ischemia [27] and ALS [14].

In contrast, recent studies have shown that apocynin failed to show any improvement in transgenic animal models of Alzheimer's disease [28] or ALS [29]. *In vitro* studies in dopaminergic neuronal cell lines and primary cultures also demonstrated a protective role of apocynin in 1-methyl-4-phenyl-pyridinium ion (MPP^+^) or MPTP-induced NADPH oxidase mediated apoptotic cell death [10,30]. Also, a pro-oxidative nature of apocynin has been shown in non-phagocytic cells, where it increases ROS production significantly [31]. Thus, these studies suggest that the development of an improved apocynin related compound may yield a better neuroprotective agent for treatment of PD.

In the central nervous system, glial activation involving astrocytes, microglial cells, lymphocyte infiltration, and production of proinflammatory mediators including cytokines, chemokines, prostaglandins, and reactive mediators, such as reactive nitrogen species (RNS) and ROS, are all hallmarks of inflammatory reactions. MPP^+^, the active metabolite of MPTP, is believed to be responsible for glial activation mediated inflammation and neurodegeneration [2]. In our study, we also observed marked activation of microglia and astrocytes, measured by Western blotting and immunohistochemistry after MPTP treatment in SN, and diapocynin significantly attenuated MPTP-induced microgliosis and astrogliosis (Figure 2).

Nuclear factor kappa B (NF-κB), a transcription factor, has been shown to be an important regulator of the microglial and astroglial proinflammatory reactions in the SN. The promoter regions of proinflammatory molecules, including iNOS, contain the binding sites for NF-ĸB [19]. Astroglia and microglia in the healthy brain do not express iNOS, but following toxic or inflammatory damage, reactive astroglia and microglia express iNOS in the brain [32]. Studies have shown that MPTP treatment produces significantly reduced neuronal loss in mice deficient in iNOS compared to their wild-type counterparts [33].

In this study, we demonstrate that diapocynin, a pharmacological inhibitor of microglial NADPH oxidase, effectively attenuates MPTP-induced increases in iNOS expression (Figure 3), suggesting the potential use of diapocynin as an anti-inflammatory agent. RNS as well as ROS play a pivotal role in oxidative stress and inflammation in PD. NADPH oxidase, which is a major ROS-producing enzyme of microglial cells, mediates superoxide production and controls the levels of pro-inflammatory neurotoxic factors, such as TNFα and IL-1β [34]. In our study, we demonstrate that diapocynin attenuates MPTP-induced expression of microglial gp91phox in SN and thereby reduces the production of ROS (Figure 4).

Besides having direct toxic effects on nigral dopaminergic neurons, nitric oxide (^·^NO) and superoxide O_2_^−^ derived from astrocytes and microglia can react to form the highly reactive nitrogen species peroxynitrite (ONOO^-^). Peroxynitrite causes nitration of tyrosine residues in various proteins including TH and α-synuclein [21,35]. Peroxinitrite mediated nitration of TH is associated with reduced enzymatic activity.

3-NT is widely used as a marker of nitrative damage. Here, we found increased expression of 3-NT in dopaminergic neurons in SN of MPTP-treated mice, predominantly co-localized with TH-positive dopaminergic neurons (Figure 5A,B,C). However, diapocynin significantly decreased the MPTP-induced increase in 3-NT in dopaminergic neurons in the SN.

Along with peroxynitrite, levels of 4-HNE, an unsaturated aldehyde generated during lipid peroxidation, were also significantly increased in the SN of PD brains compared to controls [36]. 4-HNE has been demonstrated to block mitochondrial respiration and induce caspase-dependent apoptosis [37,38]. In our study, we showed increased expression of 4-HNE, a marker of oxidative damage in the SN of MPTP-treated mice, which was colocalized in the cytosol of TH-positive dopaminergic neurons (Figure 5D,E,F). However, diapocynin significantly decreased the amount of 4-HNE in the MPTP-treated SN, indicating that diapocynin acted by attenuating ROS generation.

The lack of an effective therapy to halt the progression of PD has been a longstanding challenge in the field. Administration of a dopamine agonist or levodopa has been the leading treatment for PD symptoms, but these treatments do not affect disease progression. Various putative neuroprotective agents, including glial cell line-derived neurotrophic factor (GDNF), brain-derived neurotrophic factor (BDNF), TGF-β and other small molecule compounds, have been tested in animal models of PD [39,40]. However, most of these compounds failed in either pre-clinical trials or human trials due to their inability to cross the blood-brain barrier or due to limited bioavailability. Moreover, they also caused adverse side effects. Hence, understanding the mechanism of the disease process and development of a successful neuroprotective therapeutic approach to halt the disease progression are of principal importance in PD research.

Diapocynin has several advantages compared to other experimental drugs, including its parent compound apocynin. These include: (1) co-treatment of diapocynin and MPTP profoundly attenuated MPTP-induced glial activation and proinflammatory events, (2) diapocynin suppressed oxidative stress *in vivo* in the SN of MPTP-treated mice, (3) diapocynin treatment improved MPTP-induced behavioral deficits, (4) diapocynin protected TH-positive dopaminergic neurons from MPTP toxicity and restored the level of dopamine and its metabolites, and (5) oral administration of diapocynin on day 4, after the disease has been initiated by MPTP, also restored the levels of striatal dopamine neurotransmitters in MPTP-treated mice, suggesting that diapocynin could attenuate disease progression.

It is noteworthy that diapocynin does not interfere with MPTP metabolism, demonstrating the true neuroprotective effect in the MPTP model. Also, diapocynin is fairly nontoxic, as the mice treated with diapocynin alone (300 mg/kg/day) for 12 days did not show any sign of behavioral imparities and their neurotransmitter levels were not different from the saline-treated control mice (Figures 1E,F,G,H and 8D,E,F). Another advantage is that diapocynin can be administered orally by gavage. Being a lipophilic molecule, diapocynin easily crosses the blood-brain barrier and enters the SN (>1.5 μg/mg tissue) and striatum (>0.9 μg/mg tissue) regions of brain, as detected by LC/MS/MS (Figure 1C and D). We had to use 300 mg/kg oral dose in order to achieve a desired neuroprotective effect. Although the exact reason for the requirement of a high dose of diapocynin is not clear, it is possible that diapocynin rapidly undergoes metabolic degradation similar to apocynin [41]. Nevertheless, future studies are needed to clarify the metabolic fate of diapocynin *in vivo*. Taken together, our results demonstrate that diapocynin is a promising neuroprotective agent that deserves further exploration for its use in clinical settings.

## Conclusions

In summary, our results demonstrate that oral administration of diapocynin, a metabolite of apocynin, attenuates key neuroinflammatory events, including microglial and astroglial activation, iNOS upregulation, and oxidative and nitrative damage, in a MPTP mouse model of PD. Importantly, diapocynin treatment protects against nigral dopaminergic neuronal damage and behavioral deficits in the animal model of PD. This systematic characterization of the anti-inflammatory and neuroprotective efficacy of diapocynin in pre-clinical models of PD will facilitate further exploration of the compound for clinical application in the future.

## Abbreviations

3-NT: 3-nitrotyrosine; 4-HNE: 4-hydroxynonenal; ALS: Amyotrophic lateral sclerosis; DAB: 3,3'-diaminobenzidine; DOPAC: 3,4-dihydroxyphenyl-acetic acid; FJB: Fluoro-Jade B; GFAP: Glial fibrillary acidic protein; HPLC: High-performance liquid chromatography; HVA: Homovanillic acid; Iba-1: Ionized calcium binding adaptor molecule 1; iNOS: Inducible nitric oxide synthase; MPP^+^: 1-methyl-4phenyl-pyridinium; MPTP: 1-methyl-4-phenyl-1,2,3,6-tetrahydropyridine; MS: Mass spectrometry; PD: Parkinson’s disease; PFA: Paraformaldehyde; RNS: Reactive nitrogen species; ROS: Reactive oxygen species; SN: Substantia nigra; TH: Tyrosine hydroxylase.

## Competing interests

The authors declare that they have no competing interests.

## Authors’ contributions

AG and AGK designed research. AG, JJ and PS performed research. AG and AGK analyzed data. AG, BK, AGK wrote the paper. AG, AK, VA, BPD, BK and AGK were involved in editing drafts of the manuscript. All authors approved the final manuscript.

## Supplementary Material

Additional file 1 **Figure S1.** Diapocynin protects dopaminergic neurons in the MPTP model of PD. Mice were administered diapocynin (300 mg/kg/day) by oral gavage 24 h before MPTP treatment, and co-treatment with MPTP (25 mg/kg/day) continued for 5 days, and post-treatment with MPTP (25 mg/kg/day) continued for 6 days. Control mice received 10% ethanol in saline. Seven days after the last MPTP injection, mice were sacrificed and substantia nigra (SN) sections were processed for TH. **(A)** Double labeling of tyrosine hydroxylase (TH) and Fluoro-Jade B (FJB) in SN sections. **(B)** TH-DAB pictures were captured at 10× magnification and TH and FJB double labeled pictures were captured at 20× magnification. Click here for file
