# Supplementary figures and images for: Anti-inflammatory and neuroprotective effects of an orally active apocynin derivative in pre-clinical models of Parkinson’s disease
Source: J Neuroinflammation. 2012 Oct 23;9:241. doi: 10.1186/1742-2094-9-241 (PMC3488558; doi:10.1186/1742-2094-9-241)

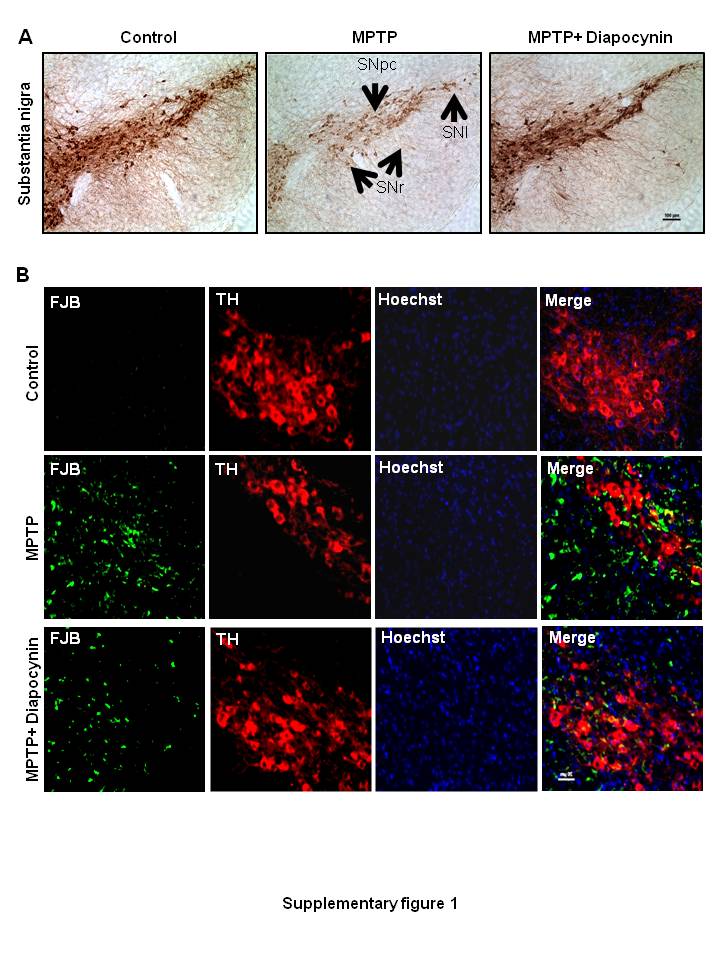

Supplement: Additional file 1 — Figure S1. Diapocynin protects dopaminergic neurons in the MPTP model of PD. Mice were administered diapocynin (300 mg/kg/day) by oral gavage 24 h before MPTP treatment, and co-treatment with MPTP (25 mg/kg/day) continued for 5 days, and post-treatment with MPTP (25 mg/kg/day) continued for 6 days. Control mice received 10% ethanol in saline. Seven days after the last MPTP injection, mice were sacrificed and substantia nigra (SN) sections were processed for TH. (A) Double labeling of tyrosine hydroxylase (TH) and Fluoro-Jade B (FJB) in SN sections. (B) TH-DAB pictures were captured at 10× magnification and TH and FJB double labeled pictures were captured at 20× magnification. [file 1742-2094-9-241-S1.jpeg]
